# Personalized treatment of brain metastases: Evolving survival prediction models may benefit from evaluation of serum tumor markers (narrative review)

**DOI:** 10.3389/fonc.2022.1081558

**Published:** 2022-12-01

**Authors:** Carsten Nieder, Nicolaus H. Andratschke, Anca L. Grosu

**Affiliations:** ^1^ Department of Oncology and Palliative Medicine, Nordland Hospital, Bodø, Norway; ^2^ Department of Clinical Medicine, Faculty of Health Sciences, UiT – The Arctic University of Norway, Tromsø, Norway; ^3^ Department of Radiation Oncology, University Hospital Zurich, University of Zurich, Zurich, Switzerland; ^4^ Department of Radiation Oncology, Medical Center, Medical Faculty, University Freiburg, Freiburg, Germany

**Keywords:** brain metastases, prognostic model, score, biomarkers, tumor markers

## Abstract

Treatment of a limited number of brain metastases (oligometastases) might include complex and sometimes invasive approaches, e.g. neurosurgical resection followed by post-operative stereotactic radiotherapy, and thus, correct identification of patients who are appropriate candidates is crucial. Both, staging procedures that visualize the true number of metastastic lesions and prognostic assessments that identify patients with limited survival, who should be managed with less complex, palliative approaches, are necessary before proceeding with local treatment that aims at eradication of all oligometastases. Some of the prognostic models, e.g. the LabBM score (laboratory parameters in patients with brain metastases), include blood biomarkers believed to represent surrogate markers of disease extent. In a recent study, patients with oligometastases and a LabBM score of 0 (no abnormal biomarkers) had an actuarial 5-year survival rate of 27% after neurosurgical resection and 39% after stereotactic radiotherapy. Other studies have tied serum tumor markers such as carcinoembryonic antigen (CEA) to survival outcomes. Even if head-to-head comparisons and large-scale definitive analyses are lacking, the available data suggest that attempts to integrate tumor marker levels in blood biomarker-based survival prediction models are warranted.

## Introduction

**Figure 1:**
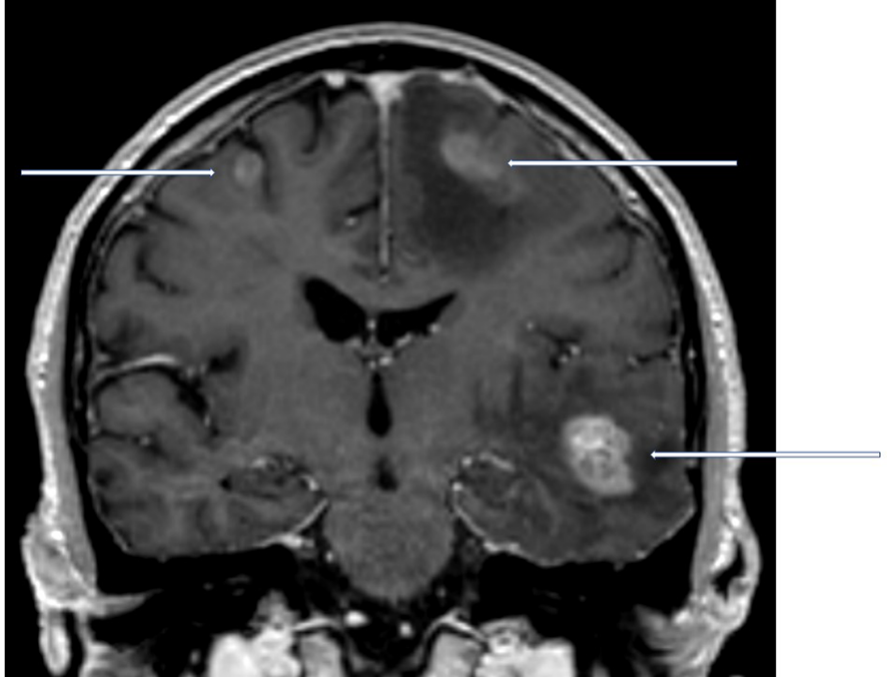
Coronal contrast-enhanced T1 weighted magnetic resonance imaging scan of a newly diagnosed patient with non-small cell lung cancer and 3 synchronous brain metastases. In the hypothetical presence of an additional adrenal gland metastasis, this patient would still be considered oligometastatic.

Brain metastases often develop as a component of widespread cancer dissemination in the terminal phase of the disease, when survival is limited to just few months (1–4). Nevertheless, a minority of patients present with clearly distinct tumor burden, meeting the current definition of oligometastatic disease, i.e. 1-5 lesions (5–7). As a result of both favorable tumor biology and effective treatment approaches, long-term survival can be achieved in patients with brain oligometastases (**Figure 1**) (8). Neither previous treatment of extracranial oligometastases nor presence of simultaneous, limited extracranial spread precludes long-term survival. Given that treatment might include complex and sometimes invasive approaches, e.g. neurosurgical resection followed by post-operative stereotactic radiotherapy, correct identification of patients who are appropriate candidates is crucial (9). Both, staging procedures that visualize the true number of metastastic lesions and prognostic assessments that identify patients with limited survival, who should be managed with less complex, palliative approaches, are necessary before proceeding with local treatment that aims at eradication of all oligometastases. Palliative approaches include supportive measures, corticosteroids, whole-brain radiotherapy (WBRT) and, if believed to represent the least toxic short-course radiotherapy regimen, in selected patient’s stereotactic radiosurgery.

**Figure 2:**
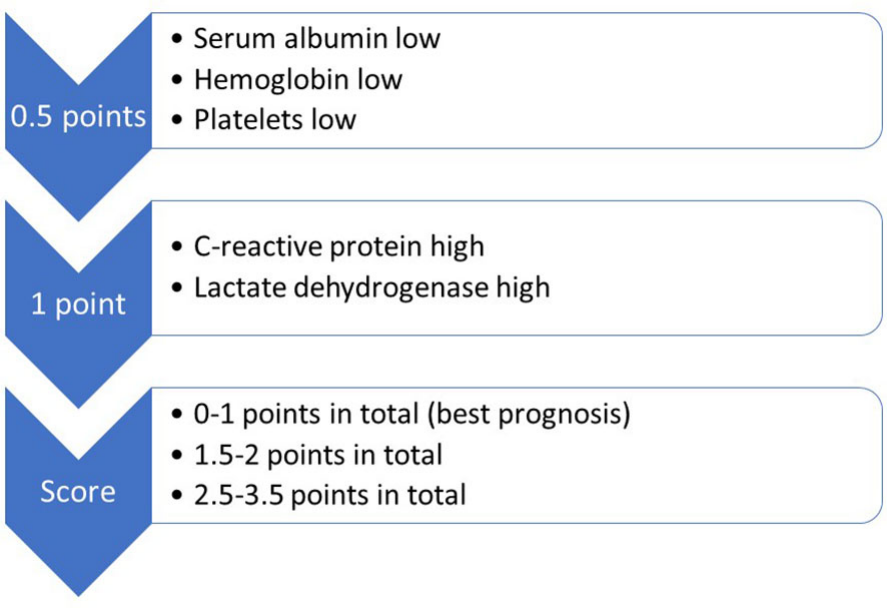
The LabBM score predicting overall survival in patients with brain metastases.

Prognostic assessment has long incorporated patient- and disease-related factors, e.g. performance status, age and number of brain metastases (10, 11). The gradually evolving models have also been tailored to cancer biology, e.g. in non-small cell lung (NSCLC) and breast cancer, where alterations that can be targeted with systemic anti-cancer drugs (epidermal growth factor receptor (EGFR) mutations etc.) influence assignment to one of three or four prognostic strata, depending on the score used in clinical practice. Some of the prognostic models include blood biomarkers believed to represent surrogate markers of disease extent, e.g. serum lactate dehydrogenase (LDH) and C-reactive protein (CRP) (12, 13). The validated LabBM score (laboratory parameters in patients with brain metastases) incorporates five simple, inexpensive blood tests (**Figure 2**), which might be helpful when trying to counterbalance limitations of routine imaging studies (14, 15). For example, if a patient with three small brain metastases from a previously resected clear cell kidney cancer presents with computed tomography (CT) imaging reports suggesting the absence of extracranial metastases and locoregional relapse, while the blood tests show abnormal LDH (1 point according to the LabBM score), CRP (1 additional point according to the LabBM score) and hemoglobin (plus 0.5 points according to the LabBM score; sum 2.5 out of maximum 3.5 points according to the LabBM score; other parameters: low albumin, low platelets), false negative imaging results may be suspected. The expected survival of a patient with 2.5 points is much shorter than that of a patient with 0 points. As recently discussed, even correct imaging findings are not always easy to classify, because consensus on how to count involved organs is still lacking (16). Replacing the number of involved organs, which has been shown to impact prognosis (17), with a surrogate such as the LabBM score might facilitate consistent classification.

## Published studies leading to the hypothesis

Publications were identified through a systematic search of the National Library of Medicine’s PubMed database (January 2000 - September 2022) with combinations of the key words brain or cerebral or central nervous system metastases or metastasis, secondary brain tumor, tumor marker, carcinoembryonic antigen (CEA), CA15-3 and CA-125. Data from our own previous studies will be provided to set the stage before reviewing other publications.

A recent retrospective study of 101 patients with maximum 4 brain metastases and 5 metastases in total (21% had limited extracranial metastases) showed that 49% had normal blood test results (LabBM score 0 points) (18). These patients’ median survival of 23 months was significantly longer than that of patients with higher LabBM score. In a multivariable model, LabBM score, performance status and single brain metastasis were associated with significantly better survival. Limited extracranial metastases did not worsen prognosis. Patients with LabBM score 0 had an actuarial 5-year survival rate of 27% after neurosurgical resection and 39% after stereotactic radiotherapy. Therefore, larger confirmatory studies are warranted to conclusively define the added value of blood biomarkers as part of the decision making in patients with oligometastases who are candidates for aggressive local treatment.

**Figure 3:**
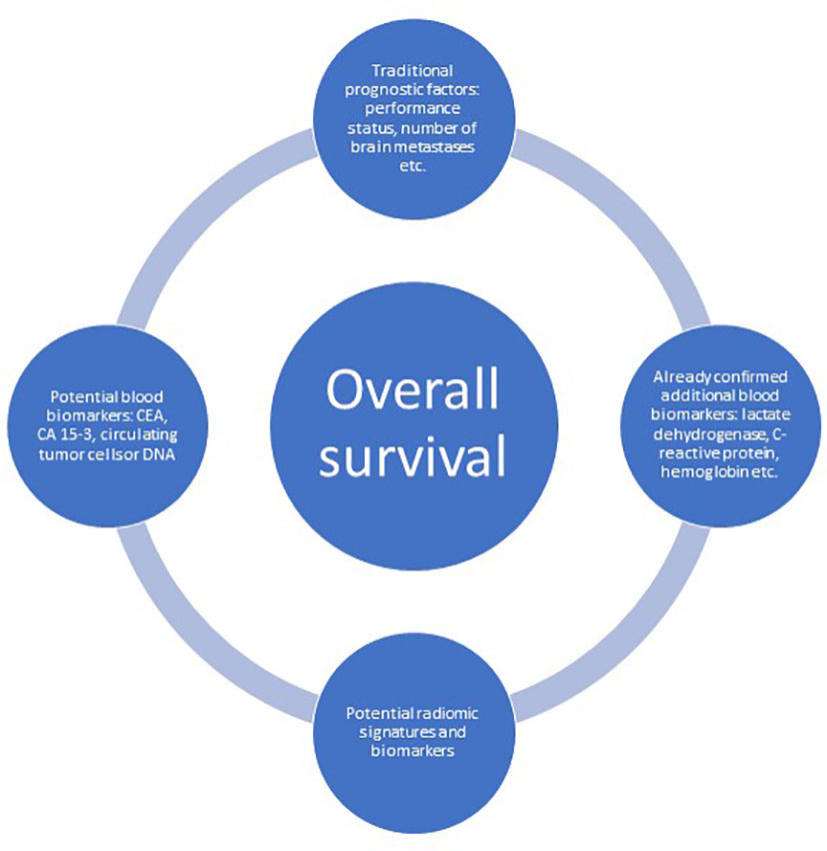
The evolving landscape of baseline, not-treatment-related prognostic factors predicting overall survival in patients with brain metastases.

Biomarkers such as LDH and CRP represent just a limited sample among a considerable number of possible cancer-associated laboratory abnormalities. So-called tumor markers might also reflect the severity of disease or the overall tumor burden, including disseminated small deposits, which typically escape radiological imaging (**Figure 3**). A previous retrospective analysis included 120 patients with known LDH and albumin treated with WBRT in two different situations: 1) brain metastases detected at initial cancer diagnosis (n = 46) and 2) brain metastases at later time points (n = 74) (19). Twenty-six patients (57%) from group 1 had at least one tumor marker analyzed, and 11 patients (24%) had abnormal results. Twenty-two patients (30%) from group 2 had at least one tumor marker analyzed, of whom 16 patients (22%) had abnormal results. Overall, an additional marker was found in 36% of patients with normal LDH and albumin, i.e. components of the LabBM score. Regarding CEA in colorectal cancer with brain metastases, the marker positivity rate was 80% in that study. The corresponding figure was 79% for CA 15-3 in breast cancer with brain metastases. In the latter group, CA 15-3 values above median predicted shorter survival (median 1.9 vs. 13.8 months, p=0.1). The group sizes were too small to provide sufficient statistical power, perform multivariable analyses or draw definitive conclusions.

In a recent Japanese study with 53 patients with lung or breast cancer and intracranial metastases, only 15 patients (28%) did not show elevated serum tumor marker levels (20). These numbers were higher than those reported previously by our own group (19). Irrespective of inter-study differences, the proportion of patients with elevated tumor markers is high enough to warrant additional investigation. Furthermore, retrospective studies have suggested that tumor markers contribute to survival prediction models. Koo et al. analyzed 106 colorectal patients undergoing WBRT with or without surgery and/or boost radiotherapy for brain metastases at three institutions (21). Older age (>65 years), multiple brain metastases (≥3), elevated level of CEA (>5 ng/ml) at diagnosis of brain metastases, and extracranial metastases were adverse prognostic factors for overall survival. Patients with 0-1 factor showed better 1-year survival (77%) than patients with 2 factors (17%) or 3-4 factors (4%; p<0.001). In a different study, Noura et al. found that the prognosis of patients with brain metastases from colorectal cancer was associated with the number of involved (metastatic) organs, and the serum CEA level (22).

Additional evidence was provided by Wei et al. who studied 66 EGFR mutation-positive NSCLC patients with brain metastases treated with WBRT and targeted drugs (23). In the survival analysis, age, CEA and status of the primary tumor were significant prognostic factors. They defined 3 patient groups with significantly different survival times: group I, age <65 years and CEA ≤10 µg/ml; group II, age <65 years and CEA >10 µg/ml or age ≥65 years and CEA ≤10 µg/ml; and group III, age ≥65 years and CEA >10 µg/ml. Iwasaki et al. reported on 41 patients who underwent lung surgery plus brain surgery, a strategy resulting in 3-year survival of 23% (24). The 3-year survival of patients with high CEA was 0 vs. 40% for those with normal CEA. Their multivariate Cox model identified both adenocarcinoma histological subtype, node status and high serum CEA as independent prognostic factors. A comparable study was performed by Kanou et al. (25). These patients with NSCLC and synchronous brain metastases were treated with surgical resection, too. Multivariate analysis demonstrated that CEA level, primary tumor size, and the presence of lymph node involvement were predictive of overall survival (all p<0.05).

Divine et al. studied patients with brain metastases from gynecological malignancies (26). On univariate analysis, primary ovarian disease, CA-125 <81 U/mL at brain metastases diagnosis, and isolated versus multi-focal metastases were all associated with longer survival. Isolated brain metastasis remained the only significant predictor on multivariate analysis, however the study size provided limited statistical power, comparable to our previous analysis in breast cancer (19).

## The hypothesis and its future evaluation

We hypothesize that tumor markers such as CA 15-3 may be relevant biomarkers with potential application in the setting of oligometastases considered for surgical resection and/or stereotactic radiotherapy. In a first step, retrospective patient cohorts with available blood test results can be analyzed. As a standard, LDH, CRP, albumin, hemoglobin and platelets (all components of the LabBM score) must be available and in addition, the added value of a given tumor marker is studied in a multivariable model. For colorectal cancer, the model may include CEA and the 5 components of the LabBM score. For each primary tumor type with available tumor marker, a separate analysis is performed. Following the principles of the LabBM score, which dichotomizes each blood test result (normal vs. abnormal), assessment of tumor markers is added. However, it would also be interesting to study different cut-offs, e.g. 1.5- or 2-times upper limit of normal. If warranted, a second step would be added, consisting of prospective data collection and analysis.

## Discussion

Promising results were seen in a recent study of 101 patients with maximum 4 brain metastases and 5 metastases in total, with respect to the fact that those with LabBM score 0 had an actuarial 5-year survival rate of 27% after neurosurgical resection and 39% after stereotactic radiotherapy (18). Utilization of the score, or blood biomarkers in a broader sense, renders comprehensive radiological re-staging unnecessary and is a low-cost intervention. Implementation of the LabBM score in clinical routine is feasible and has the potential to improve decision making and prediction of the long-term survival probability. However, it appears prudent to study modifications of the score, which may increase its ability to mirror a patient’s true cancer burden. In this context, tumor markers such as CEA, CA15-3 or CA-125 are considered potentially relevant additions to the 5 blood tests already included. Even if large-scale definitive analyses are lacking, the available data suggest that attempts to integrate tumor marker levels in blood biomarker-based survival prediction models are warranted. These models are increasingly relevant in the present era of improved systemic anti-cancer treatment, which already has shown a positive impact on survival (27).

## Data availability statement

The original contributions presented in the study are included in the article/supplementary material. Further inquiries can be directed to the corresponding author.

## Author contributions

CN, NA and AG drafted the manuscript and read and approved the final manuscript.

## Funding

Open Access funding provided by UiT The Arctic University of Norway. The publication charges for this article have been funded by a grant from the publication fund of UiT The Arctic University of Norway.

## Conflict of interest

The authors declare that they have no known competing financial interests or personal relationships that could have appeared to influence the work reported in this paper.

## Publisher’s note

All claims expressed in this article are solely those of the authors and do not necessarily represent those of their affiliated organizations, or those of the publisher, the editors and the reviewers. Any product that may be evaluated in this article, or claim that may be made by its manufacturer, is not guaranteed or endorsed by the publisher.
